# Modeling and Simulation of Network-on-Chip Systems with DEVS and DEUS

**DOI:** 10.1155/2014/982569

**Published:** 2014-04-17

**Authors:** Michele Amoretti

**Affiliations:** Centro Interdipartimentale SITEIA.PARMA, Università degli Studi di Parma, Parco Area delle Scienze 181a, 43124 Parma, Italy

## Abstract

Networks on-chip (NoCs) provide enhanced performance, scalability, modularity, and design productivity as compared with previous communication architectures for VLSI systems on-chip (SoCs), such as buses and dedicated signal wires. Since the NoC design space is very large and high dimensional, evaluation methodologies rely heavily on analytical modeling and simulation. Unfortunately, there is no standard modeling framework. In this paper we illustrate how to design and evaluate NoCs by integrating the Discrete Event System Specification (DEVS) modeling framework and the simulation environment called DEUS. The advantage of such an approach is that both DEVS and DEUS support modularity—the former being a sound and complete modeling framework and the latter being an open, general-purpose platform, characterized by a steep learning curve and the possibility to simulate any system at any level of detail.

## 1. Introduction

Efficient on-chip communication is a primary factor in the performance of large core-count systems. Indeed, the research community has directed substantial attention to* networks on-chip (NoCs)*, which use packet-switched networks for communications within large VLSI systems on-chip (SoCs) [1]. NoCs provide enhanced performance, scalability, modularity, and design productivity as compared with previous communication architectures, such as buses and dedicated signal wires.

In a NoC system, modules like processor cores, memories, and specialized IP blocks exchange data using a network as a “public transportation” subsystem for the information traffic. A NoC is constructed from multiple point-to-point data links interconnected by* switches*, such that messages can be relayed from any source module to any destination module over several links, by making routing decisions at the switches. Such a definition based on switches is usually interpreted, so that a single shared bus, a single router, or a point-to-point networks are not NoCs, but practically all other topologies are. A NoC is similar to a modern telecommunications network, using digital bit-packet switching over multiplexed links.

The NoC design space is very large and high dimensional. It includes the optimization of topology, routing mechanism, congestion control methodologies, link capacities, number of buffers and virtual channels per link, and others. Thus, there is an increasing need for effective tools for rapid performance analysis. Current NoC evaluation methodologies rely heavily on analytical modeling and simulation. Unfortunately, there is no standard modeling framework. Moreover, the available simulators use proprietary configuration languages, are hardly portable, and are usually not interoperable with other tools. To cover the existing and future NoC diversities, NoC simulators should be more modular, scalable, extendible, and fully parametric.

To cope with these issues, Ahmadinejad et al. proposed a NoC model [2] based on the Discrete Event System Specification (DEVS) [3], which is widely recognized as a sound and complete modeling framework, with a large community of researchers and practitioners. Ahmadinejad et al. implemented their NoC model in DEVS-Suite (http://devs-suitesim.sourceforge.net), a parallel DEVS simulator with support for (i) automating design of experiments in combination with (ii) animating models and (iii) generating data trajectories at run-time. Our approach is different, as we choose a general-purpose simulation environment, namely, DEUS (http://code.google.com/p/deus/) [4]; we provided it with DEVS support; then, we used it to simulate DEVS-based NoC models. DEUS is characterized by a steep learning curve and the possibility to simulate highly dynamic complex systems—such as peer-to-peer networks, markets, virtual resources in data centers, cellular automata, and others—at an any level of detail. Moreover, it is provided with a set of visual tools for the design, configuration, and analysis of parallel parametric simulations. The advantage of using DEUS is that it allows for memory-efficient dynamic reconfiguration of the simulated model.

The paper is organized as follows. In Section 2, we present the state of the art in NoC simulation. In Section 3, we recall the DEVS formalism. In Section 4, we summarize the features of the DEUS simulator, focusing on the recently added DEVS support. In Section 5, we illustrate an example of modular DEVS-based NoC model and its simulation with DEUS. Finally, in Section 6, we conclude the paper and also propose future work.

## 2. State of the Art

Most NoC simulators are written in C++ or System C (a system description language based on C++), while few of them are written in Java (which produces less efficient but more portable code, with respect to C++ and System C). None of the simulation tools described in the following—which are those that are currently developed/maintained—used a widely accepted modeling framework, for which it is almost impossible to develop a model with one tool, and then used it with another tool.

BookSim was initially developed by Dally and Towles, for their seminal book about interconnection networks [5]. The current version (released in 2010) is BookSim 2.0 (http://nocs.stanford.edu/cgi-bin/trac.cgi/wiki/Resources /BookSim), which supports a wide range of topologies such as mesh, torus, and flattened butterfly networks, provides diverse routing algorithms, and includes several options for customizing the network's router microarchitecture. BookSim is written in C++ and uses a LEX and YACC generated parser to process the configuration file that must be passed to the simulator. Each simulation has three basic phases: warm-up, measurement, and drain. The length of the warm-up and measurement phases is a multiple of a basic sample period. The current latency and throughput (rate of accepted packets) for the simulation are printed after each sample period. The overall throughput is determined by the lowest throughput of all the destinations in the network, but the average throughput is also displayed. Most of the simulator's components are designed to be modular, so tasks such as adding a new routing algorithm, topology, or router microarchitecture should not require a complete redesign of the code.

TOPAZ (http://code.google.com/p/tpzsimul/) is a general-purpose interconnection network simulator which allows modelling a wide variety of message routers (from buffered crossbar to rotary router), with different trade-offs between speed and precision [6]. The design of the tool is object oriented and its implementation is in C++ language. In TOPAZ, each network is constructed hierarchically. The simulator builds the network and the links (topology), then the network builds all the routers, and finally the routers build its intrinsic components and interconnect them. Each simulated structure is associated with two C++ classes: components and flows. The components are descriptive, characterizing each structure and its relationship with the remaining components of the system. The flows establish how the stream of the information will move inside the component. As an example, for a buffer structure, the component will determine its size, number of ports, or delay, while the flow will determine its behavior according to the flow control selected. During the running phase, all system components are iteratively visited and all dependent flows are simulated. While TOPAZ is a time-driven simulation tool, some flows can internally be constructed as finite state machines, making these components event driven. The simulator supports parallel execution using standard POSIX threads.

The NOXIM simulator (http://noxim.sourceforge.net/) is developed using System C and provides a command line interface for defining several parameters of a NoC. In particular, the user can customize the network size, buffer size, packet size distribution, routing algorithm, selection strategy, packet injection rate, traffic time distribution, traffic pattern, and hot-spot traffic distribution. The simulator allows NoC evaluation in terms of throughput, delay, and power consumption. Such information is delivered to the user both in terms of average and per-communication results. In detail, the user is allowed to collect different evaluation metrics, including the total number of received packets/flits, global average throughput, max/min global delay, total energy consumption, per-communications delay/throughput/energy, and others [7].

NIRGAM (http://nirgam.ecs.soton.ac.uk/) is a System C based discrete event, cycle accurate NoC simulator. It provides substantial support to experiment with NoC design in terms of routing algorithms and applications on various topologies. NIRGAM models a NoC as a 2-dimensional interconnection of tiles. Each tile consists of a core connected to a router/switch by a bidirectional core channel. A tile is connected to neighbor tiles by bidirectional channels. Configurable NoC parameters are the topology size (*m* × *n*), the clock frequency, the buffer depth, the flit size, and the virtual channels. Currently supported routing algorithms are only *XY*, odd even, and source.

HNOCS (http://hnocs.eew.technion.ac.il) is an open source implementation of a NoC simulation framework using OMNeT++. As an event driven simulation engine, OMNeT++ provides C++ APIs to a rich set of services that can be used to model, configure, describe the network topology, collect simulation data, and perform analysis [8]. HNOCS supports heterogeneous NoCs with variable link capacities and number of VCs per each unidirectional port. Heterogeneous NoCs [9] offer better performance compared to homogeneous NoCs, since SoCs and CMPs are heterogeneous in terms of module-to-module traffic requirements.

Few simulators are specially designed for being executed on parallel/multicore architectures. One of them is Graphite (http://groups.csail.mit.edu/carbon/?page_id=111), which provides high performance for fast design space exploration and software development. Its high efficiency is balanced by the lack of portability: it is written in C++ but, basically, it works only with Debian Linux. HORNET (http://csg.csail.mit.edu/hornet/) is also written in C++, and requires the Boost C++ library and Python 2.5. The HORNET NoC system model is composed of a number of interconnected tiles. Each tile comprises a processing element (PE), which can be a MIPS CPU simulator or a script-driven injector or a Pin front-end, a bridge that converts packets to flits, and, finally, the network switch node itself. Since each tile can be run in a separate thread, intertile communication is synchronized using fine-grained locks. To avoid unnecessary synchronization, each tile has a private independently initialized Mersenne Twister random number generator and collects its own statistics; at the end of the simulation, the per-tile statistics are collected and combined into whole-system statistics [10].

## 3. DEVS Modeling

DEVS [3] is a formalism which allows representing any system having a finite number of changes in a finite interval of time. In that way, systems modeled by Petri Nets, State Charts, Event Graphs, and even Difference Equations can be seen as particular cases of DEVS models.

In its most general definition, a DEVS (atomic) model is a structure

(1) $$\begin{array}{c} M=\langle X,S,Y,\delta_{\mathrm{int}},\delta_{\text{ext}},\lambda ,t_{a}\rangle , \end{array}$$

where

- *X*  is the set of input values,
- *S* is a set of states,
- *Y* is the set of output values,
- *δ*_int⁡_ : *S* → *S* is the internal transition function,
- *δ*_ext_ : *Q* × *X* → *S* is the external transition function, where *Q* = {(*s*, *e*) | *s* ∈ *S*, 0 ≤ *e* ≤ *t*_*a*_(*s*)} is the total state set (*e* is the time elapsed since last transition),
- *λ* : *S* → *Y* is the output function,
- *t*_*a*_ : *S* → *R* ^+^ is the time for which the system stays in state *s* if no external event occurs.

The external transition function dictates the system's new state when an external event occurs. Such a state is determined by the input (*x*), the current state (*s*), and how long the system has been in that state (*e*).

The input trajectory is a series of external events affecting the system. The state trajectory is affected by external events in *X* but also by internal events. The output trajectory depicts the output events that are produced by the output function just before applying the internal transition function at internal events.

Functions *δ*_int⁡_, *δ*_ext_, *λ*, and *t*_*a*_ can be either deterministic or stochastic.

**Figure 1:**
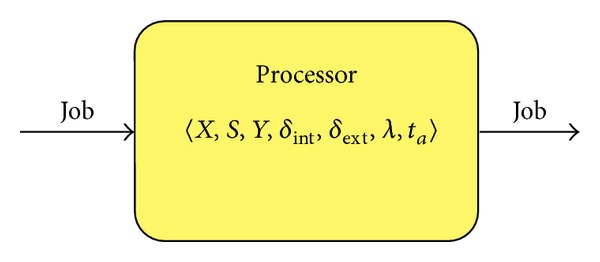
DEVS model of a processor.

For example, a simple DEVS model for a processor (Figure 1) is the following:

(i) *X* = *R*,
(ii) *S* = {“idle”, “busy”} × *R* ^+^ × *R*,
(iii) *Y* = *R*
(iv) *δ*_int⁡_(phase, *σ*, job) = (“idle”, *σ*, job),
(v) (2) δext(phase,σ,job,e,x) ={(“busy”, PTx,x)if  phase=“idle”(“busy”, PT  job−e, job)if  phase=“busy”
(vi) *λ*(“busy”, *σ*, job) = job
(vii) *t*_*a*_(phase, *σ*, job) = *σ*,

where *R* is the set of jobs the processor can receive and generate, *σ* is the time the processor stays in one of the two possible phases, that is, “idle” or “busy,” and *PT*_job_ is the processing time needed to execute a job. The internal transition function is evaluated each time a job is done. The external transition function is evaluated each time a new job is received by the processor. If the processor is idle, the new job *x* is acquired and executed, for which the state of the processor changes to “busy” and the remaining time in such state *σ* is set to *PT*_*x*_. If the processor is busy, the incoming job is discarded and the remaining time in current state is updated to *PT*_job_ − *e*.

DEVS modeling is made easier with the introduction of input and output ports, by re-defining *X* and *Y* as follows.

- *X* = {(*p*, *v*) | *p* ∈ InPorts, *v* ∈ *X*_in_} is the set of input ports and values.
- *Y* = {(*p*, *v*) | *p* ∈ OutPorts, *v* ∈ *Y*_out_} is the set of output ports and values.

*DEVS coupled models* are built from components that are specified as DEVS models. The specification of DEVS coupled models, in case of DEVS with ports, is the following:

(3) $$\begin{array}{c} N=\langle X,Y,D,\left\{M_{d}d\in D\right\},\text{EIC},\text{EOC},\text{IC},\text{Select}\rangle , \end{array}$$

where

- *X* is the set of input ports and values,
- *Y* is the set of output ports and values,
- *D* is the set of component names,
- for each *d* ∈ *D*, *M*_*d*_ is a DEVS model,
- EIC is the external input coupling that connects external inputs to component inputs,
- EOC is the external output coupling that connects component outputs to external outputs,
- IC is the internal coupling that connects component outputs to component inputs,
- Select : 2^*D*^ − { } → *D* is the tie-breaking function (used to serialize imminent component actions).

In DEVS coupled models, no direct feedback is allowed.

**Figure 2:**
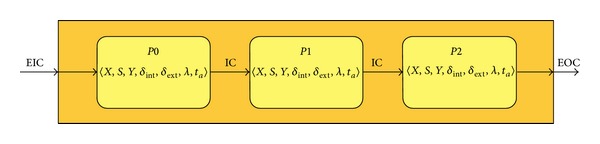
DEVS coupled model of a pipeline made by three processors in series.

As an example, we construct a simple DEVS coupled model by placing three processors in series to form a pipeline (Figure 2). The formal specification is as follows:

- InPorts = {“in”};
- *X*_in_ = *R*;
- *X* = {(“in”, *v*) | *v* ∈ *R*};
- OutPorts = {“out”};
- *Y*_out_ = *R*;
- *Y* = {(“out”, *v*) | *v* ∈ *R*};
- *D* = {*P*0, *P*1, *P*2};
- *M*_0_ = *M*_1_ = *M*_2_ = *M*;
- EIC = {[(*N*, “in”), (*P*0, “in”)]};
- EOC = {[(*P*2, “out”), (*N*, “out”)]};
- IC = {[(*P*0, “out”), (*P*1, “in”)], [(*P*1, “out”), (*P*2, “in”)]};
- Select = *δ*_int⁡_ first.

The Select rule is important in the case of coupled processors having imminent outputs. For example, if *P*0 and *P*1 generate output at the same time, there are two possible choices for *P*1: (a) to apply *δ*_int⁡_, which would allow accepting the input coming from *P*0, or (b) to apply *δ*_ext_ first, which would result in discarding the input because of “busy” state. In this example model, the *Select* function dictates that the function to be applied first is *δ*_int⁡_.

An important subset of DEVS coupled models is constituted by parallel DEVS coupled models [3]. The parallel DEVS specification allows multiple ports to receive values at the same time, without the need of the Select.

## 4. Simulation of DEVS Models with DEUS

Discrete event simulation works by maintaining a list of events sorted by their scheduling times. Executing events results in new events being scheduled and inserted into the event list as well as events being deleted and removed from the event list. A problem that arises in discrete event simulation is that of* simultaneous events*, for which different orderings of activation result in different evolutions of the simulation. The approach employed by most simulation packages is to define a priority among the components. Zeigler et al. defined a hierarchical simulator for hierarchical DEVS coupled models, consisting of devs-simulators and devs-coordinators [3]. Shortly, each DEVS atomic model is simulated by a devs-simulator, and each DEVS coupled model is simulated by a devs-coordinator, which manages the simulators of the subcomponents. As these can be themselves DEVS coupled models, a devs-coordinator is able to manage also devs-coordinators.

The general-purpose simulation tool called DEUS [4] follows a different approach. (Although their names suggest a connection between them, DEVS and DEUS are completely independent.) Its Java API allows developers to implement (by subclassing) (i)* nodes*, that is, the entities which interact in a complex system, leading to emergent behaviors such as humans, pets, cells, robots, or intelligent agents [11, 12]; (ii)* events*, for example, node births and deaths, interactions among nodes, interactions with the environment, logs, and so on; and (iii)* processes*, either stochastic or deterministic ones, constraining the timeliness of events.

DEUS has been designed having in mind the three basic concepts listed above and no specific modeling tool at all. Nevertheless, it is possible to map DEUS concepts to DEVS ones—for example, a DEUS node can be the implementation of a DEVS model.

Once specific Java classes have been implemented, it is possible to configure a simulation with the DEUS graphical user interface, which includes the following:

- the Visual Editor, for the generation of XML documents describing the simulations;
- the Automator, for the execution of parametric simulations and the automatic generation of statistics (in a Gnuplot-compliant format).

Last but not least, DEUS supports parallel (multicore and/or distributed) simulations [13].

**Figure 3:**
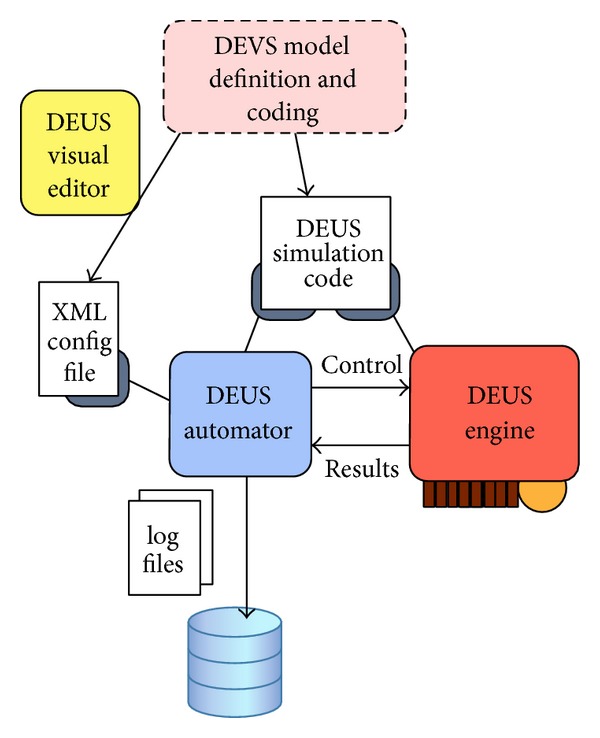
Discrete event simulation with DEUS, driven by DEVS models.

Figure 3 illustrates how DEUS simulation models, in terms of XML configuration files and Java code, are created (using also a Visual Editor) and then executed by means of the Automator and the Engine. The former allows performing sensitivity analysis, by setting ranges for node and process parameters. The Engine is the core of DEUS, managing the event queue and the simulation loop.

**Figure 4:**
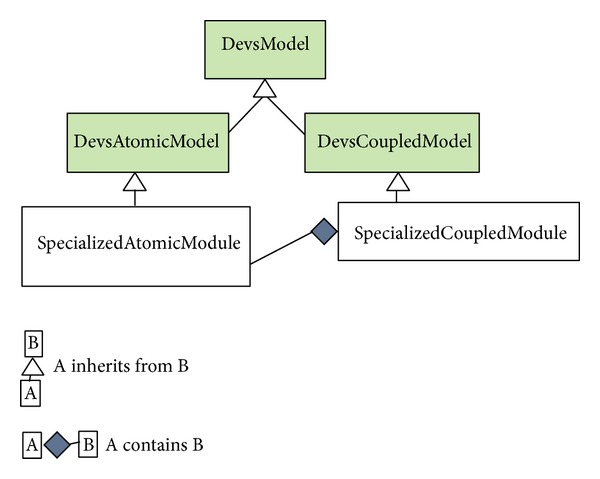
Class diagram of the package for DEVS support in DEUS we recently introduced.

A node represents a dynamic system characterized by a set of possible states, whose transition functions may be implemented either in the source code of the events associated with the node or in the source code of the node itself. Multiscale modeling of complex systems is achieved by means of connected heterogeneous nodes. DEUS comes with a library of predefined, common processes, and many others can be implemented by the user. Recently we added DEVS support, by means of a new package which includes the general-purpose classes  DevsAtomicModel and  DevsCoupledModel, both implementing the  DevsModel interface (see Figure 4). In next section, we describe a modular DEVS-based NoC model and its simulation with DEUS.

## 5. NoC Example

**Figure 5:**
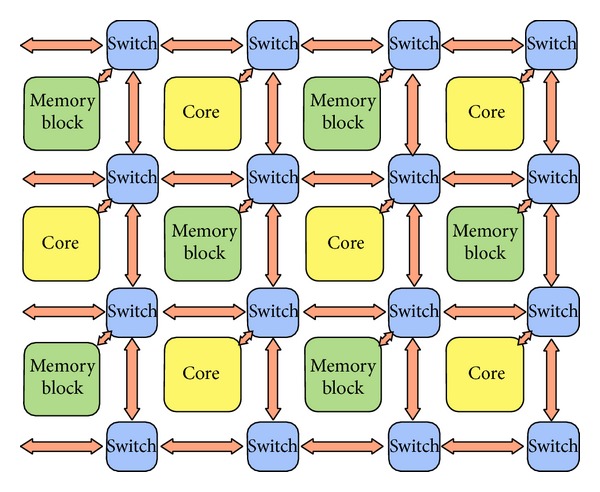
Example NoC consisting of a mesh of switches and resources (cores and memory blocks).

We considered a NoC system consisting of a mesh of switches and resources (i.e., processor cores or memory blocks), which are placed on slots formed by the switches, like the one described by Sun et al. [14] and sketched in Figure 5. We modeled resources and switches and the whole NoC, using DEVS coupled models. The mesh is an *m* × *n* grid of switches, each one being connected either to a core or to a memory block. For simplicity, resources are alternated—that is, core - memory block - core -, and so forth—on both axes.

**Figure 6:**
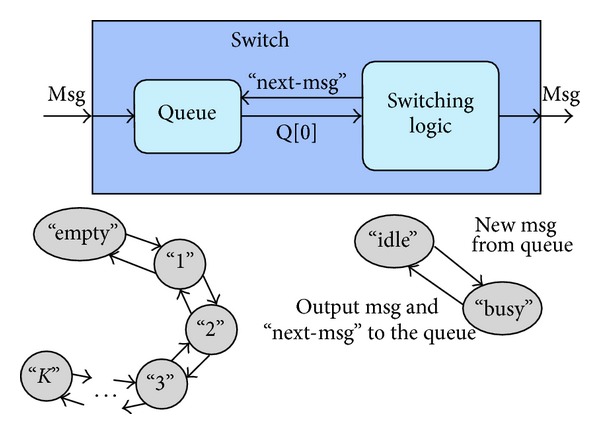
DEVS models of the switch in the proposed NoC example.

A switch is modeled as a DEVS model with input and output ports, which are coupled, respectively, with output and input ports of other switches or resources. As illustrated in Figure 6, the switch itself is a DEVS coupled model, made of two DEVS atomic models, one representing a queue and the other representing the system that implements the switching logic. To model their interaction, we use the following strategy. When the switching logic completes a job (i.e., decides the destination of a message and forwards it), it does send a “next-msg” input to the queue, to make the latter provide a new message. The DEVS model of the queue is defined as follows:

(i) *X* = *R*′;
(ii) *S* = {“empty”, “1”,…, “*K*”} × *R* ^+^ × *R*;
(iii) *Y* = *R*;
(iv) there is no *δ*_int⁡_, as the queue is a passive module;
(v) (4) δext(phase,σ,msg,e,x)={(“1”,σ,x,x) if  phase=“idle”,  x!=“next-msg”(“i+1”,σ,msg) if  phase=“i”,  i∈[1,K−1],  x!=“next-msg”(phase,σ−e,msg) if  phase=“K”,  x!=“next-msg”(“i−1”,σ,msg) if  phase=“i”,  i∈[2,K],  x=“next-msg”(“idle”,σ,ϕ) if  phase∈  {“1”,“idle”},  x=“next-msg”
(vi) $\lambda (\text{phase},\sigma ,\text{msg})=\{\begin{array}{c} \phi \;\;\text{if}\;\text{phasE}\;=\;\text{“idle”}\\ \text{msg}\;\;\text{if}\;\text{phase}\;!\;=\;\text{“idle”} \end{array}$
(vii) *t*_*a*_(phase, *σ*, msg) = *σ*,

where *R* = {“get-data”, “put-data”, “store-data”} is the set of messages that can be generated by core and memories, *R*′ = *R* ∪ {“next-msg”}, and *K* is the maximum number of messages that can be enqueued. The output message is the one stored at the head of the queue. If the queue is empty, there is no output—we use *ϕ* to represent the output in this case.

The core and the switching logic system are modeled as the processor illustrated in Section 3. The switching logic system receives incoming messages and routes them according to the *XY* routing strategy [15]—first in *x*- or horizontal-direction to the correct column and then in *y*- or vertical direction to the destination switch. In general, any specific switching algorithm can be “plugged” into the model.

**Figure 7:**
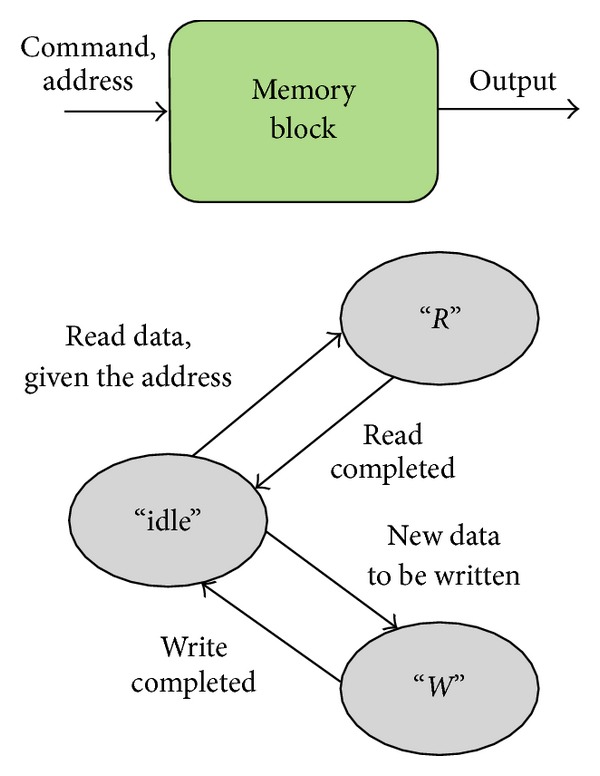
DEVS models of the memory block in the proposed NoC example.

The memory block, illustrated in Figure 7, is modeled as follows:

(i) *X* = *R* × *A*;
(ii) *S* = {“idle”, “*W*”, “*R*”} × *R* ^+^ × *R*;
(iii) *Y* = {*M*[*a*]}  ∀*a* ∈ *A*;
(iv) there is no *δ*_int⁡_, as the memory block is a passive module;
(v) (5) δext(phase,σ,data,e,address,command) ={(phase,σ−e,data) if  phase  !=“idle”(“R”,PTread, M[address]) if  phase=“idle”, command=“read”(“W”, PTwrite,ϕ) if  phase=“idle”, command=“write”
(vi) $\lambda (\text{phase},\sigma ,\text{output})=\{\begin{array}{cc} \phi \;\;\text{if}\;\text{phase}\;!=\;\text{“}R\text{”} & \\ \text{output}\;\;\text{if}\;\text{phase}=\text{“}R\text{”} & \end{array}$
(vii) *t*_*a*_(phase, *σ*, output) = *σ*,

where *R* = {“read”, “write”} is the set of commands the memory block can handle, *A* is the set of addresses, and *ϕ* means no output.

**Figure 8:**
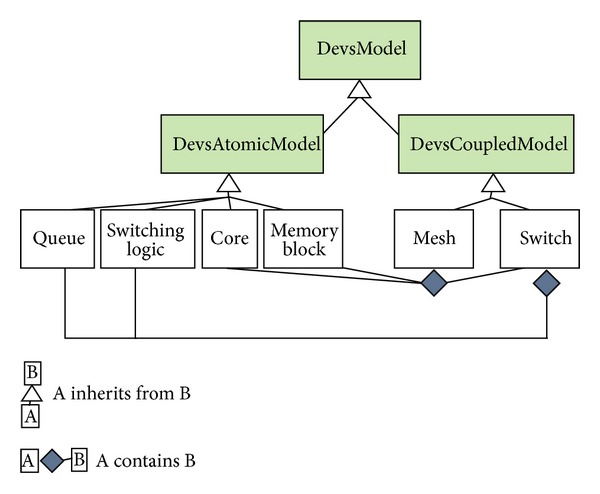
Class diagram illustrating the DEVS-to-DEUS mapping for the proposed NoC example.

To perform DEUS-based simulation, we mapped the DEVS models to the following Java classes:  Core,    MemoryBlock,  Switch (composed by  Queue and  SwitchingLogic),  Mesh. The class diagram in Figure 8 illustrates the relations between such classes, the basic classes  DevsAtomicModel and  DevsCoupledModel, and the  DevsModel interface. In the diagram, shaded classes are those provided by the  devs package we recently added to DEUS.

According to the DEUS approach, it is not necessary to explicitly model links among network nodes. Parameters like link delay and maximal bandwidth can be embedded either in the Java classes representing the resources or in the scheduling processes of events that describe the internode communications (i.e., packet delivery). For this example, we adopted the former approach.

In detail, we defined a specific  recv( ) method in every class implementing a DEVS model. Such a method implements the logic described by the *δ*_ext_ and *λ* functions. For the core, the  recv( ) method handles the following messages:

- “put-data”: the core enters the “busy” state and sends a “finish-proc” message to itself, to be received when the processing phase is completed (this is just a trick to exit the “busy” state);
- “finish-proc”: the core enters the “idle” state and sends a “store-data” message to a randomly selected memory block, with a random double ∈[0,1] as a payload.

For the memory block, the  recv( ) method handles the following messages:

- “get-data”: the memory block sends a “put-data” message to the core that issued the “get-data” message, with the mean of its stored values as a payload.
- “store-data”: the memory stores the payload of the message; if the memory is full, the less recent value is discarded, to make room for the new one.

The  recv( ) method of the switch passes the message to the same method of the queue instance, where the message is stored, if there is room—otherwise, it is dropped. After a delay which depends on the state of the queue (i.e., on the length of the queue) and on the service rate of the switching logic, the message is passed to the switching logic itself, which computes the next hop—it may be another switch or the core/memory block connected to the current switch. The total delay for a message to pass through a switch and be delivered to the next module is given by the sum of the following components:

- time spent in the queue,
- time for computing the next hop,
- transmission time.

Then, we defined a  SendMessageEvent class, whose  run( ) method just calls the appropriate  recv( ) on the message destination. Instances of  SendMessageEvent are generated as a consequence of a “new task” event (see below for details) or during a message propagation process, which always involves a couple of modules (with one exception, discussed below).

**Figure 9:**
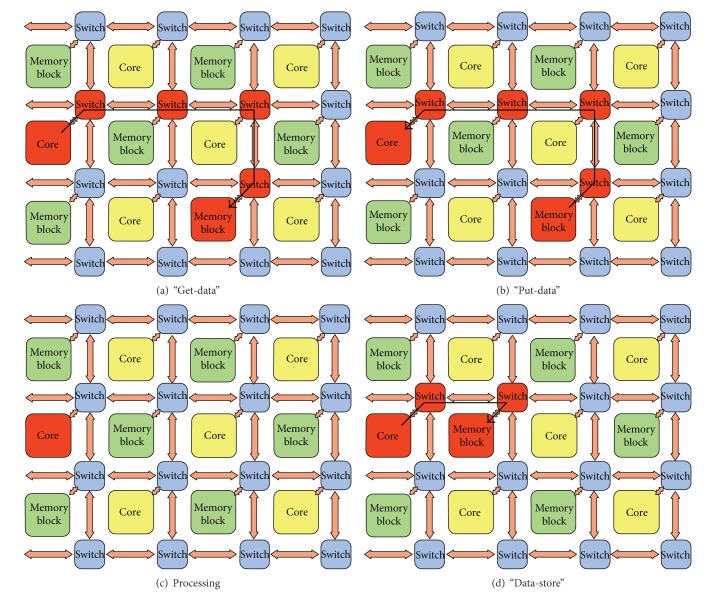
An example of the simulated interaction scenario, started by a  NewTaskEvent. For each phase, involved modules are uniformly shaded, and arrows show the information flow.

For each  NewTaskEvent, an idle core is randomly selected, to start a “get-data” message propagation (as illustrated in Figure 9, where involved modules are uniformly shaded and arrows show the information flow). Once the destination memory block has been reached, the latter module starts “put-data” message propagation towards the core which started it all. When such a core receives the “put-data” message, it enters the “busy” state. The processing task ends with a  SendMessageEvent the core schedules on itself (according to its *δ*_int⁡_ function) to enter to “idle” state and send a “data-store” message to a randomly chosen memory block. Of course, any other interaction scheme could have been implemented.

In general, such a DEVS+DEUS model and simulation allows evaluating different routing strategies (either static or dynamic) and queue management mechanisms implemented in the switches. Example parameters we may consider in this context are the* communication load* (a measure of average traffic in the network), the* packet delay* [14], and the* average throughput* of switches.

To measure variables over the simulated virtual time, specific logging events, as well as visualization events, must be implemented and periodically scheduled. For this example, we implemented a  LogNetworkStatsEvent, which computes performance indices, such as the the Hit Ratio, that is, the number of completed tasks, versus the number of issued ones.

Table 1 illustrates the configuration of the simulations we executed—with 10 runs, each one being characterized by a different seed for the random number generator.

**Figure 10:**
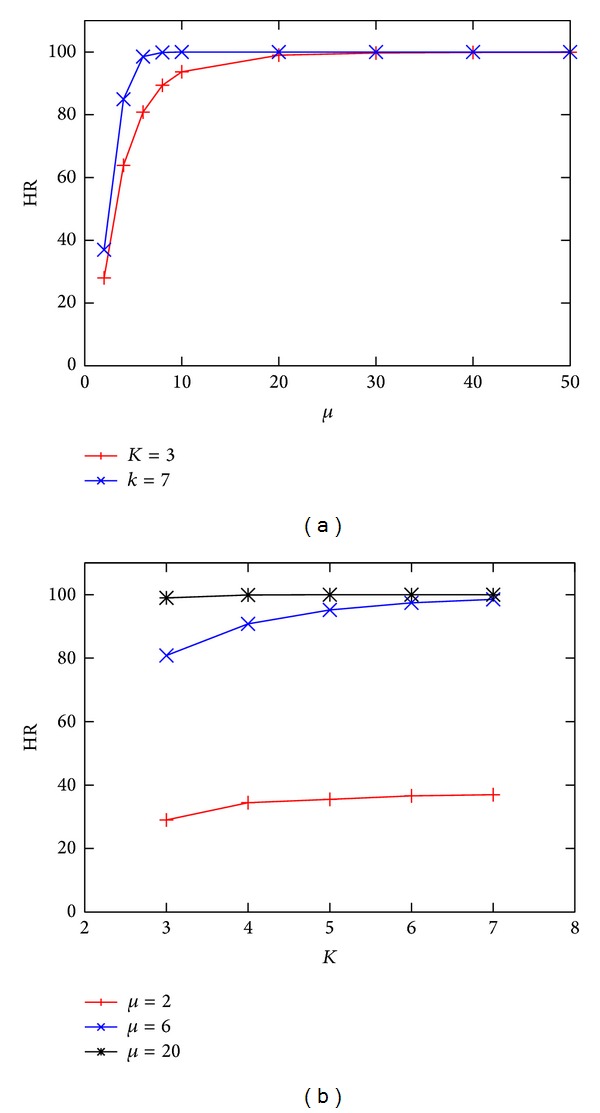
Hit Ratio as a function of *μ* (a) and *K* (b).

The task interarrival time is such that, on the average, each core starts a new task every *μ* · 8 ≥ 16 ns, which is higher than the core processing time. This is a reasonable configuration, but it is not sufficient to guarantee a 100% Hit Ratio, which is influenced by several factors—such as the max queue length *K*, the size of the mesh, and the delays of the switches and memory blocks. For a simulated system lifetime of 100 ms, we obtained the results illustrated in Figure 10, where the Hit Ratio is shown as a function of *μ* and *K*. Simulation time ranges from few seconds (when *μ* > 20) to few minutes (when *μ* < 10), on a MacBook Pro with 2.4 GHz Intel Core 2 Duo and 4 GB 1066 MHz DD3 RAM.

## 6. Conclusions

In this paper, we have illustrated how to model NoCs by means of the DEVS formalism and to simulate such models with the DEUS simulation tool. The proposed approach has two main advantages. First, DEVS and its dialects allow modelling almost any scenario, including concurrent executions. Second, the DEUS simulation environment is portable, efficient, provided with useful tools for the rapid configuration of highly automated simulations. The combination of DEVS and DEUS allows studying NoCs models with the desired level of detail and efficiently comparing different configurations of parameters.

Regarding future work, we plan to implement and share other NoC models and simulations, with different network topologies and routing algorithms. Moreover, we are interested in defining DEVS models of NoCs with Quality of Service (QoS) constraints and using DEUS to check whether such constraints are respected. QoS refers to the levels of guarantees given for data transfers. Guarantees are related to timing (min. throughput, max. latency, and max. latency jitter), integrity (max. error rate and max. packet loss), and packet delivery (in-order or out- of-order).

## Figures and Tables

**Table 1:** Experiment configuration.

Number of rows	*m* = 4
Number of columns	*n* = 4
Transmission delay	Exponential with mean value 1 ns
Switching logic delay	Exponential with mean value 5 ns
Max queue length	*K* = 3, 5, 7,…
Memory block size	*S* = 100
Memory block processing time	Uniform with max value 100 ns
Core processing time	Uniform with max value 10 ns
Task interarrival time	Exponential with mean value *μ* = 2, 4, 6,… ns
